# Photonic hook formation in near-infrared with MXene Ti_3_C_2_ nanoparticles†

**DOI:** 10.1039/d0na00485e

**Published:** 2020-09-22

**Authors:** Marat Spector, Angeleene S. Ang, Oleg V. Minin, Igor V. Minin, Alina Karabchevsky

**Affiliations:** School of Electrical and Computer Engineering, Ben-Gurion University of the Negev Beer-Sheva 8410501 Israel alinak@bgu.ac.il; National Research Tomsk State University Tomsk 634050 Russia

## Abstract

MXenes, a recently developed class of 2D materials, have attracted considerable attention because of their graphene-like but highly tunable properties. It appears that the metallic properties of MXene titanium carbide are pronounced in near-infrared with well-defined localised surface plasmon resonance (LSPR). Here, we report on a curved photonic nanojet, known as the photonic hook, applied on a titanium carbide nanoparticle for the particle's optomechanical manipulation. We show that the optical forces generated and applied on titanium carbide nanoparticles of various shapes are based on the LSPR excitation in near-infrared. We compare the obtained results to traditional plasmonic gold nanoparticles which exhibit LSPR in visible. Considering the diversity of the MXene family, this study is a first step towards photonic devices that utilize optomechanical manipulation in near-infrared for biomedical research, optical trapping and others.

## Abstract 2

This work investigates the optical forces acting on a titanium carbide nanoparticle with an incident photonic hook field.

## Graphical abstract

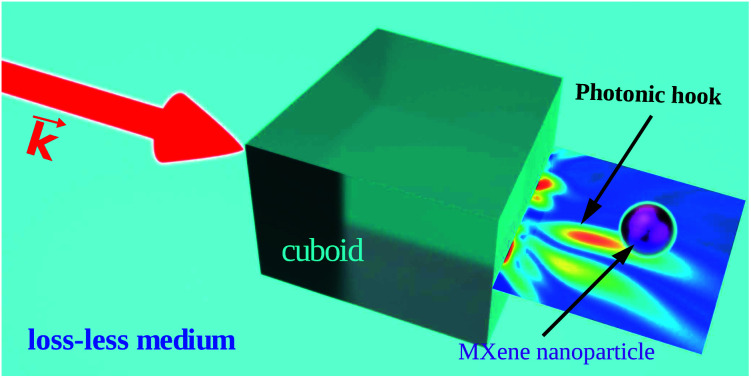

## 1 Introduction

MXenes, first discovered in 2011,^1^ are a diverse novel family of two dimensional nanomaterials formed from transition metal carbides and carbonitrides. These materials are derived from M_*n*+1_AX_*n*_, MAX phases, where *n* = 1 to 4, and M is an early transition metal produced by selectively etching the A-layers.^2^ The derived phase is labeled MXene to emphasize their graphene-like morphology.^3^ MXenes are environmentally benign, with strong hydrophilic properties due to their unique layered structure and chemical stability. These materials have demonstrated their potential as electrode materials of Li-ion batteries, supercapacitors, and sensors.^4,5^ One of the most widely studied and most promising members of the MXene materials is titanium carbide (Ti_3_C_2_). It can be readily fabricated by selectively etching aluminum from Ti_3_AlC_2_.^6^ This material is used in applications such as electrochemical capacitors,^2^ electromagnetic wave shielding,^7^ integratable photodetectors,^8^ transparent electrodes,^9^ metal ion batteries,^10^ supercapacitors,^11^ and many others.

The plasmonic properties of MXenes remain largely unexplored, but limited work on the subject exists.^12–20^ Mauchamp *et al.* revealed that Ti_3_C_2_T_*x*_ (T_*x*_ denotes surface-terminated moieties such as –O or –F) is a plasmonic material;^16^ a comprehensive study of the surface plasmons on the material has been performed at infrared (IR) wavelengths.^13^ The applications of MXene nanostructures have been investigated as a broadband absorber.^15^ Integrating MXenes into SPR sensors has significantly improved sensitivity compared to the set-up without the 2D material.^12,14^

While the studies on MXenes are focused towards their use as a 2D material, they can also exist as nanoparticles,^6,21^ flakes,^13^ and nanostructured films.^15^ In addition, the plasmonic properties of the MXene Ti_3_C_2_ can be used to enhance light–matter interactions. Among the most popular candidates for plasmonic applications is gold, due to its bulk plasmon performance in the visible range.^22^ Given sufficiently small objects, one can manipulate the movement of particles using focused laser light.^23^ Although optical manipulation is usually applied to dielectric objects, the aforementioned strong light–matter interaction arising from plasmonic resonances has been used to trap, move, rotate, and align metallic nanoparticles.^22,24–27^

One of the drawbacks of using conventional laser-based optical manipulation is the diffraction-limited trapping volume.^27^ An alternative proposed in order to achieve sub-diffraction manipulation has been explored using a high-intensity narrow light beam generated by dielectric structures, referred to as photonic nanojets.^28–32^ Photonic nanojets are usually generated using symmetric structures, usually spherical, subjected to external illumination.^28,33^ Previous work has explored a curved photonic nanojet, called the photonic hook, and its capacity for optical manipulation under continuous plane wave illumination^34^ and pulsed plane waves.^35^ It was shown that the maximum magnitude of the optical force generated by the photonic hook is near the wavelength of plasmon resonance excitation.

It should be emphasized that the metallic properties and plasmon resonance of titanium carbide are clearly defined in the near-infrared range, whereas the plasmon resonance of gold is in the visible range. This limits the application of gold in biomedical research, since its penetration depth is short. Using near-infrared wavelengths will increase the penetration depth, making it suitable for optical manipulation beneath biological layers like tissues. Moreover, optical forces on MXene particles have never been explored.

**Fig. 1:**
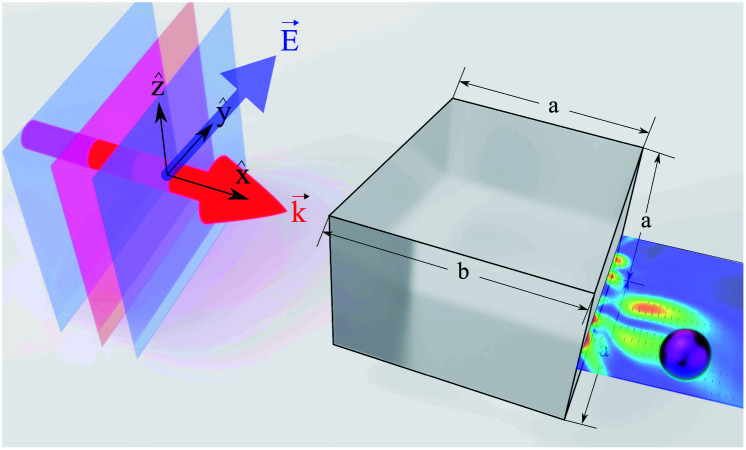
Illustration of the plane wave illuminating asymmetric auxiliary structure cuboid in the direction of vector 
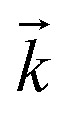
. The cuboid size parameters *a* = 3 and *b* = 4 are obtained from ref. 33.

In this paper, we report for the first time the possibility of optomechanical manipulation of Ti_3_C_2_ nanoparticles with cylindrical symmetry and movement of Ti_3_C_2_ nanospheres in a curved trajectory utilising Ti_3_C_2_ localized surface plasmon resonance (LSPR) properties in near-infrared. The studied system is shown in Fig. 1 with a plane wave illuminating the cuboid and a generated photonic hook force applied on the Ti_3_C_2_ particle. We compare our results to the magnitude trajectory of gold nanospheres moved using the photonic hook curved light force. In addition, we explore the polarizability of Ti_3_C_2_ given different particle geometries such as the nanodisk and nanorod. We will show that for the case of titanium carbide nanoparticles, the creation of the photonic hook can be successfully tuned to near-infrared.

## 2 Ti_3_C_2_ LSPR

Here we study how to manipulate a particle in near-infrared utilizing optical forces generated on Ti_3_C_2_. This effect can occur in case the localised surface plasmon is excited. At the interface where the real part of the dielectric function changes sign, such as a metal–dielectric interface, the electron oscillations occur.^36–42^ Confining the surface plasmon in a nanoparticle to a size smaller than the wavelength of light used to excite the plasmon produces a localized surface plasmon.^43,44^ This results in the enhancement of electric fields near the particle surface, and the absorption of the particle is maximized at the plasmon resonant frequency.

We compare two plasmonic materials: the commonly used gold and the novel MXene Ti_3_C_2_. The dielectric functions of gold^45^ and titanium carbide^46^ are included in the ESI file.† As opposed to gold nanoparticles, whose LSPR is located in the visible range, the location of LSPR in the case of Ti_3_C_2_ is in near-infrared.

The spectral location of the LSPR depends on the geometry of the plasmonic nanostructure and material^43,47^ and is directly related to the following expression

1 *ε*_m_(*λ*) + *Gε*_d_(*λ*) = 0;

where *ε* is the dielectric function of the metallic and dielectric substrate denoted by m and d respectively and *G* is the geometric form factor which depends on the symmetry of the considered system. For instance, for spherical nano-particles *G* = 2,^48^ while for a layered structure, *G* = 1.^49^ This factor is important as we compare different types of nanoparticles made of gold and Ti_3_C_2_ and embedded in various lossless dielectric media. Specifically, we consider different types of geometries with cylindrical symmetry such as a sphere, a needle, and a disk.

We investigate the excitation of LSPR under continuous wave (CW) illumination. Physically, this means that we do not take into account any heating effects which has to be taken into account for the case of a Gaussian beam. While the dependence of the dielectric function of gold on the temperature of either electrons or the lattice is very well known^45,50^ and was in detail discussed in our previous article,^51^ there is still a lack of experimental data regarding the dependence of the dielectric function of MXenes on the temperature of either electrons or the lattice. Consequently, we focus on comparison between MXenes and gold nanoparticles under CW illumination. Furthermore, we assume that the incident intensity is very low, meaning that we can neglect the dependence of the dielectric function of MXenes on temperature,^52,53^ which is directly related to photon absorption in near-infrared.

## 3 Nanosphere

Assuming that the radius of the nanosphere is much smaller than the incident wavelength, the polarizability of the nanosphere is given by^54^2
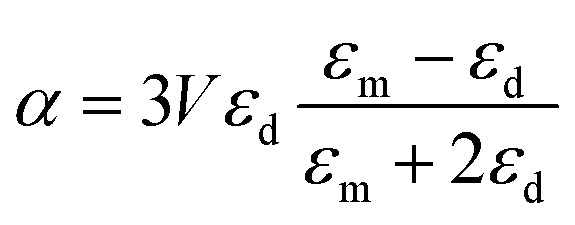
where *V* is the volume of the nanoparticle and *ε* is the dielectric function of either a dielectric or a metal, denoted by the subscript d, m, respectively.

**Fig. 2:**
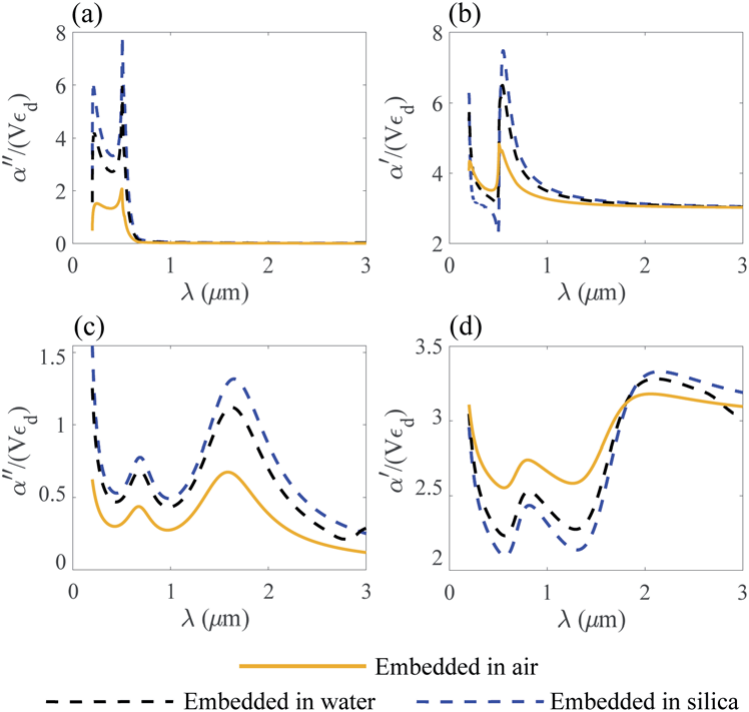
Nanosphere polarizability spectrum in air, water and silica: (a) *α*′′ of gold; (b) *α*′ of gold; (c) *α*′′ of Ti_3_C_2_; (d) *α*′ of Ti_3_C_2_.

The normalized polarizability 
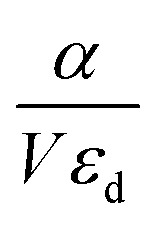
 of gold and Ti_3_C_2_ nanospheres embedded in different lossless media such as air, water or silica is presented in Fig. 2. The dispersion of the dielectric function of water and silica is based on the experimental data,^55,56^ while the dielectric function of air is assumed to be independent of the incident wavelength of the laser beam.

Fig. 2 shows that the location of LSPR is tuned to near-infrared for the case of Ti_3_C_2_ nanoparticles as compared to gold. Specifically, Fig. 2c and d show that the polarizability of Ti_3_C_2_ nanospheres attains its maximum (minimum) for the imaginary (real) part when the incident wavelength is *λ* ≈ 1.5 μm. We also note that the gold nanoparticle is a linear function of the studied media in which the nanoparticle is embedded, *i.e.*, the real and imaginary parts of the polarizability increase as the refractive index of the surrounding medium increases. In comparison, the polarizability of Ti_3_C_2_ in this region shows a very different behavior. The imaginary part of the polarizability increases as the medium changes from air to water, while the real part decreases. Analysis shows that both the materials used as embedded media show a negligible wavelength dependence of the dielectric function. Thus, the unusual behavior of the Ti_3_C_2_ nanoparticle polarizability is strictly related to the optical properties of Ti_3_C_2_.

## 4 Optical force applied on a Ti_3_C_2_ nanosphere

We analyze the optical force acting on Ti_3_C_2_ and gold nanoparticles, with the photonic hook illumination system shown in Fig. 1. The optical force *F* and the acceleration *a* acting on the nanoparticle are given according to the following expressions^57^3a
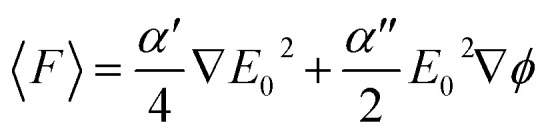
3b
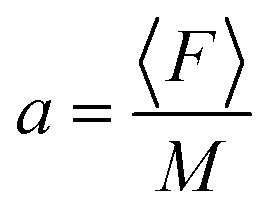
where *ϕ* is the phase and *E*_0_ is the applied electric field, and *M* is the mass of the nanoparticle. The first term of eqn (3a) is known as the gradient force, responsible for trapping (or repelling) the probe particle at locations where the field intensity gradient is high. The second term is the scattering force, which moves in the direction of the electric field propagation and is responsible for unrestricted particle motion.

**Fig. 3:**
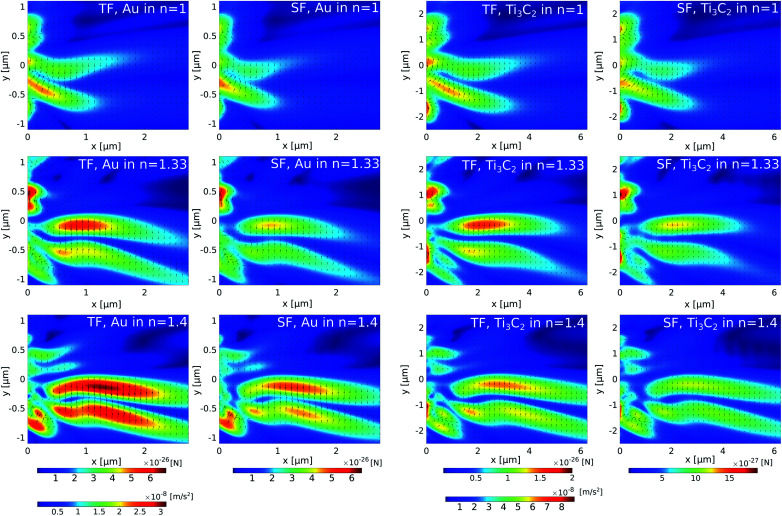
Total force and total scattering forces in (first row) air, (second row) water, and (third row) a material with *n* = 1.44. The first and second columns respectively show the total force (TF) and scattering force (SF) applied on a gold nanoparticle illuminated by *λ* = 625 nm; the third and fourth columns respectively show the total force (TF) and the scattering (SF) force applied on a Ti_3_C_2_ nanoparticle illuminated by *λ* = 1500 nm.

Assuming that the radius of the nanoparticle is *R* = 30 nm and the density *ρ* = 2 g cm^−3^ of Ti_3_C_2_,^58^ the optical force is presented for the case of gold and the Ti_3_C_2_ nanosphere embedded in different media in Fig. 3. The size of the nanoparticle is kept constant as changing the particle size would only change the magnitude of the optical force.

The cuboid, as illustrated in Fig. 1, is used to generate the photonic hook. It is composed of a cube with an attached wedge. The wedge provides the asymmetry required to generate the asymmetric photonic jet. For the photonic jet/hook to be generated, there needs to be a specific material contrast between the cuboid and the surrounding environment. It is known that an illuminated cuboid embedded in air^34,35^ creates a hook for *n*_cuboid in air_ = 1.4, corresponding to fused silica. For an illuminated cuboid embedded in other media, the material needs to be adjusted in order to preserve the material contrast. Specifically, the effective refractive index of the cuboid in the medium for the cases of a cuboid embedded in water with an index of *n* = 1.44 is 1.862 and 2.016, respectively.

For the simulation, we built a model using commercial software Lumerical finite-difference time-domain method (FDTD). Using the scattered field formalism with an initial plane wave propagating along *x* and the electric field polarized along *y*, we obtain the photonic hook field by illuminating the cuboid. The mesh was refined along *x* and *y* to have a size of 5 nm. We define the shadow side of the cuboid at *x* = 0. The field data were then transferred to Matlab where a script using the central difference method was used to calculate the field derivatives needed to obtain the optical force fields.

The analysis of Fig. 3 shows that different media have a negligible effect on the maximum optical force values. Instead, it affects the shape of the optical force. Moreover, the comparison of optical forces applied on different nanoparticles shows that the magnitude of the total optical force applied on a gold nanosphere is higher than the magnitude of the total optical force applied on a Ti_3_C_2_ nanosphere. However, due to the lower density of the Ti_3_C_2_ nanoparticle, its acceleration is approximately higher by an order of magnitude. Thus, we conclude that the Ti_3_C_2_ nanosphere can be successfully used for either trapping or moving nanoparticles when the incident wavelength is in near-infrared.

However, the spherical symmetry of the nanoparticles is an idealization of the problem. In general, the particles are not spherical and different types of geometry should be considered. To highlight the differences between the gold and Ti_3_C_2_ nanoparticles, we analyse the non-spherical geometry of the nanoparticles in the following section.

## 5 Non-spherical particles

We assume that generating the nanojet is not related to the polarizability of the nano-particle. The outcome of the nanojet which is the optical force acting on the nanoparticle depends on the geometry of the nanoparticle and its dielectric function. Here, we consider several types of nanoparticles assuming that their polarizability which is strictly related to the optical force acting on the nanoparticle can be calculated based on Rayleigh approximation. In this section, we analyze the effect of non-spherical symmetry on the polarizability *α* of the studied Ti_3_C_2_ and gold nanoparticles, which in turn affects the optical force, as given by eqn (3), acting on the nanoparticle. We study the nanodisk geometry with the radius much larger compared to the height (*h*/*r* ≪ 1). Here, we calculate the nanoodisk of height 2 nm and radius of 30 nm. For the nanorod, we assume (*r*/*h* ≪ 1). Specifically, we calculate the nanorod with a radius of 2 nm and length of 30 nm.

The most obvious extension of nanospheres is the ellipsoidal geometry. In this case, the polarizability depends on the direction of the electric field. The polarizability component in the direction of the *i*-th axis of the ellipsoid, with three orthogonal axes, is given by^59^4
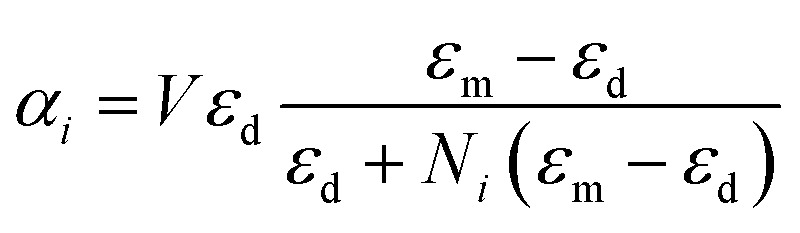
where *N*_*i*_ is the depolarization factor of the ellipsoid. The depolarization factor is a measure of whether the internal field within the ellipsoid is weakened by the polarization or not. For zero depolarization, the internal field is preserved the same as the external field. The sum of the depolarization factors for any ellipsoid is equal to 1.^59^

Assuming the semi-axes of the ellipsoid in the three orthogonal directions *a*_*x*_, *a*_*y*_ and *a*_*z*_, respectively, the depolarization factor *N*_*i*_ related to orthogonal directions is given by5
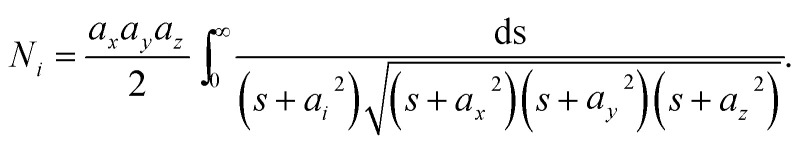


Due to the rotational symmetry of the sphere, spherical particles have three equal depolarization factors, *i.e.*, 
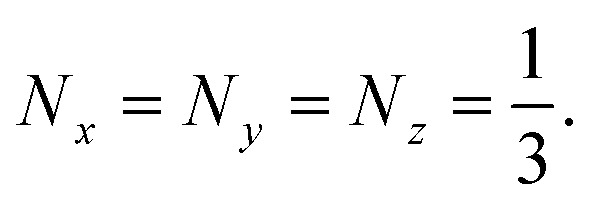


In Section 3, gold and Ti_3_C_2_ nanospheres embedded in different lossless media were analyzed. In the following section, we consider two limiting cases of ellipsoidal geometry, namely a disk, whose depolarization factors are given by (1 0 0) and a needle (0 1/2 1/2).

### 5.1 Nanodisk

**Fig. 4:**
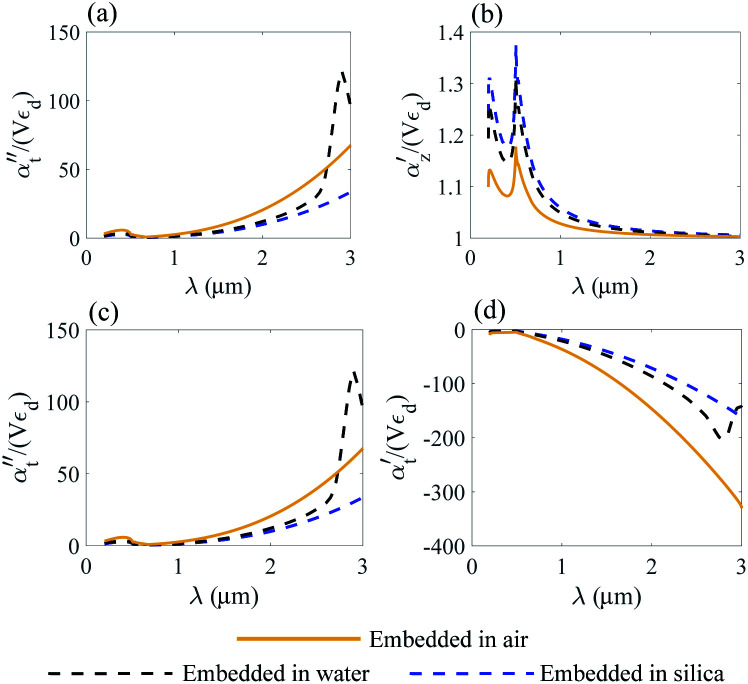
Normalized polarizability of the gold nanodisk embedded in different media. Longitudinal *α*_*z*_ (a and b) and transverse *α*_*t*_ (c and d) components are shown.

For the case of the nanodisk, the polarizability is given by^60^6a
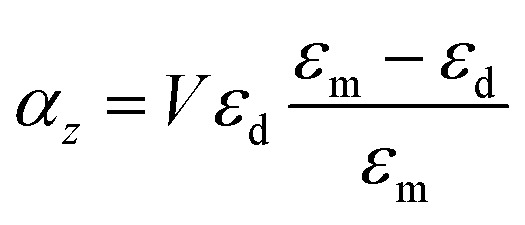
6b
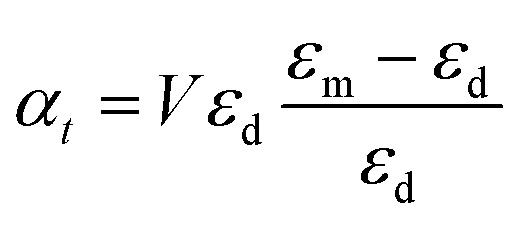
where *α*_*z*_ and *α*_*t*_ stand for the longitudinal and transverse components of polarizability with respect to the axis of the disk. The normalized polarizability of either gold or the Ti_3_C_2_ nanodisk is shown in Fig. 4.

**Fig. 5:**
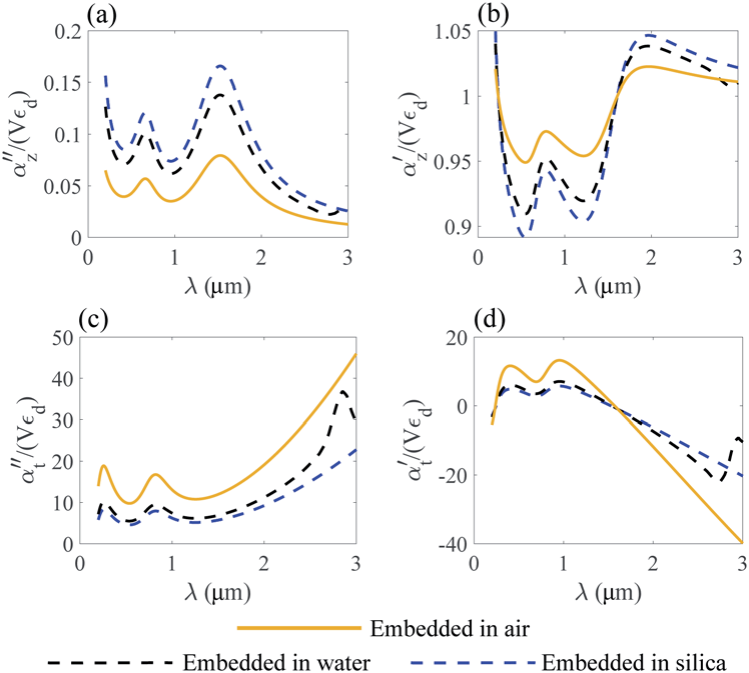
Normalized polarizability of the Ti_3_C_2_ nanodisk embedded in different media. Longitudinal *α*_*z*_ (a and b) and transverse *α*_*t*_ (c and d) components are shown.

We compare the polarizability of gold and Ti_3_C_2_ nanodisks embedded in different loss-less media. From an analysis of Fig. 4a and b, we note that for the case of gold the real and imaginary parts of *α*_*z*_ attain their maximum values in visible. These maximum values are directly related to the interband transitions in gold. For the case of the Ti_3_C_2_ nanodisk, we note that, according to Fig. 5a and b, the maximum values of the real and imaginary parts of *α*_*z*_ are obtained in near-infrared. Moreover, the case is much more complicated for the case of the transverse part of the nanoparticle polarizability. As can be concluded from Fig. 5c and d, it plausible to use the gold disk in near-infrared, assuming that the electric field is polarized along the transverse direction of the nanodisk.

We conclude that due to the higher absolute values of the real and imaginary parts of *ε* of gold in near-infrared, the electric field will decay much faster from the gold nanodisk as compared to Ti_3_C_2_. Thus, the application of the Ti_3_C_2_ nanodisk is more preferable as compared to gold. Moreover, Fig. 5b shows that the real part of polarizability of the Ti_3_C_2_ nanodisk 
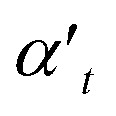
 changes its sign in a narrow spectral range. In general, the real part of polarizability is related to the gradient force which is responsible for trapping the nanoparticle. Thus, using a wideband source with a wide enough spectral range, trapping the Ti_3_C_2_ nanodisk along the *z*-axis can be accomplished.

As opposed to the nanosphere, the polarizability of either the gold or Ti_3_C_2_ nanodisk shows quite a peculiar behavior depending on the media in which these particles are embedded. Fig. 4 shows the calculated polarizability for the case of a gold nanodisk. From Fig. 4a, we note that the imaginary part of the longitudinal polarizability increases as the index of the medium increases from *n* = 1 (air) to *n* = 1.33 (water), while the real part (Fig. 4b) shows a different behavior as compared to the spherical particle. For the case of Ti_3_C_2_, both the real and the imaginary parts of the polarizability show a very different behavior when the medium changes from air to water as compared to the Ti_3_C_2_ nanosphere. This difference is associated with the geometry of the particle, while the behaviour of the spherical particle is different from that of the disk. Specifically, according to Fig. 5b, the real part of the longitudinal and transverse polarizability for the Ti_3_C_2_ nanodisk embedded in air or water is identical. This means that both air and water have the same effect on the photonic hook generation applied on titanium carbide nanoparticles.

### 5.2 Nanorod

For the case of a very thin nanorod, the polarizability is given as^60^7a
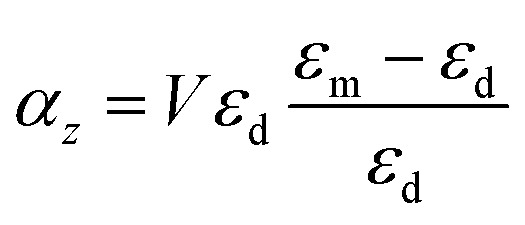
7b
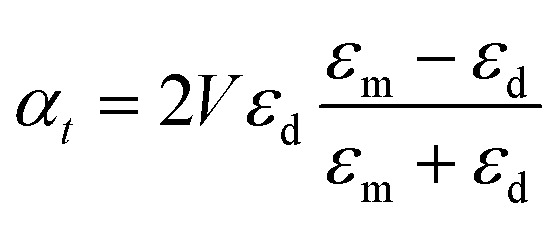


We note that according to eqn (6) and (7), the longitudinal part of the dielectric polarizability of a thin nanorod is similar to the transverse part of a disk polarizability. Thus, the analysis performed in Section. 5.1 for the case of *α*_*t*_ can be utilized in the current section.

**Fig. 6:**
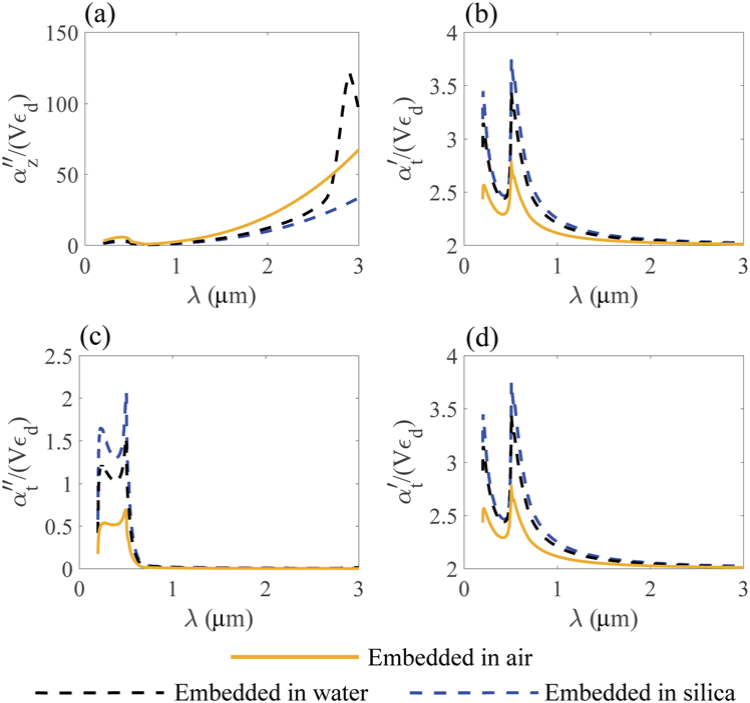
Normalized polarizability of a gold nanorod embedded in different media. Longitudinal *α*_*z*_ (a and b) and transverse *α*_*t*_ (c and d) components are shown.

**Fig. 7:**
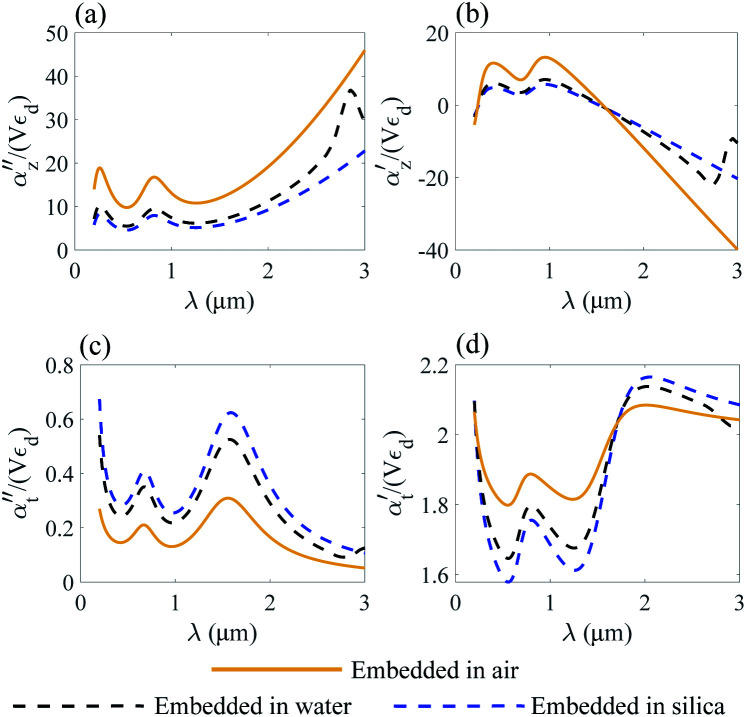
Normalized polarizability of a Ti_3_C_2_ nanorod embedded in different media. Longitudinal *α*_*z*_ (a and b) and transverse *α*_*t*_ (c and d) components are shown.

We analyze the transverse part of dielectric polarizability *α*_*t*_ of gold and Ti_3_C_2_ thin rods as shown in Fig. 6c, d and in Fig. 7c, d.

Following the same approach as in previous section, we perform a similar analysis for the case of the transverse part of the polarizability of a thin nanorod. Thus, we conclude that the application of a Ti_3_C_2_ thin nanorod is more preferable as compared to gold. In addition, based on the results of Fig. 7d, we note that trapping of a Ti_3_C_2_ nanorod can be done using a wide enough light source.

Moreover, due to different geometry of the nanoparticle the transverse part of the Ti_3_C_2_ rod polarizability shows a different behaviour as compared to the spherical and disk geometry. Specifically, the imaginary part of the polarizability 
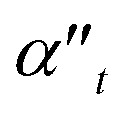
 decreases as the index of the medium increases from air to water, and the real part 
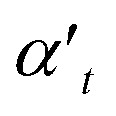
 shows a more complicated behavior. Specifically, we show that at an appropriate wavelength the real part of *α*_*t*_ is similar in all dielectric media, meaning that the media have no influence on the optical force here.

## 6 Summary

To summarise, we reported for the first time the effect of Photonic hook formation in near-infrared applied on MXene nanoparticles. To analyse this new effect, we explored different geometries of nanoparticles made of gold and titanium carbide for the generation of a photonic hook optical force. We showed that in comparison to plasmonic nanoparticles made of gold, experiencing LSPR in visible, the LSPR of Ti_3_C_2_ nanoparticles can be tuned to the near-infrared region. These findings are particularly important for biomedical applications for which the longer wavelengths of near-infrared radiation, as compared to visible, penetrate cells and tissues without any harmful rupture. Our findings are aimed at stimulating further research of Ti_3_C_2_ nanoparticles in the field of optomechanical manipulation in near-infrared and their applications in photonics, plasmonics, biomedical research and others.

## Conflicts of interest

There are no conflicts to declare.

## Supplementary Material

NA-002-D0NA00485E-s001
