# Correction: Photonic hook formation in near-infrared with MXene Ti_3_C_2_ nanoparticles

**DOI:** 10.1039/d0na90059a

**Published:** 2020-12-07

**Authors:** Marat Spector, Angeleene S. Ang, Oleg V. Minin, Igor V. Minin, Alina Karabchevsky

**Affiliations:** School of Electrical and Computer Engineering, Ben-Gurion University of the Negev Beer-Sheva 8410501 Israel alinak@bgu.ac.il; National Research Tomsk Polytechnic University Tomsk Russia

## Abstract

Correction for ‘Photonic hook formation in near-infrared with MXene Ti_3_C_2_ nanoparticles’ by Marat Spector *et al.*, *Nanoscale Adv.*, 2020, **2**, 5312–5318, DOI: 10.1039/D0NA00485E

The authors regret that one of the affiliations (affiliation b) was incorrectly shown in the original manuscript. The corrected list of affiliations is as shown above.

The Royal Society of Chemistry apologises for these errors and any consequent inconvenience to authors and readers.

## Supplementary Material

