# Supplementary material for: Photonic hook formation in near-infrared with MXene Ti3C2 nanoparticles
Source: Nanoscale Adv. 2020 Sep 22;2(11):5312–8. doi: 10.1039/d0na00485e (PMC9418953; doi:10.1039/d0na00485e)
Supplement: NA-002-D0NA00485E-s001 [file NA-002-D0NA00485E-s001.pdf]

Electronic Supplementary Material (ESI) for Nanoscale Advances.
This journal is © The Royal Society of Chemistry 2020

Journal Name

ARTICLE TYPE

Cite this: DOI: 00.0000/xxxxxxxxxx

Photonic hook formation in near-infrared with MXene Ti_3C_2 nanoparticles: Supplementary Figure

Marat Spector,[a] Angeleene S. Ang,[a] Oleg V. Minin,[c,d] Igor V. Minin,[c,d] and Alina Karabchevsky[‡a]

Received Date
Accepted Date

DOI: 00.0000/xxxxxxxxxx

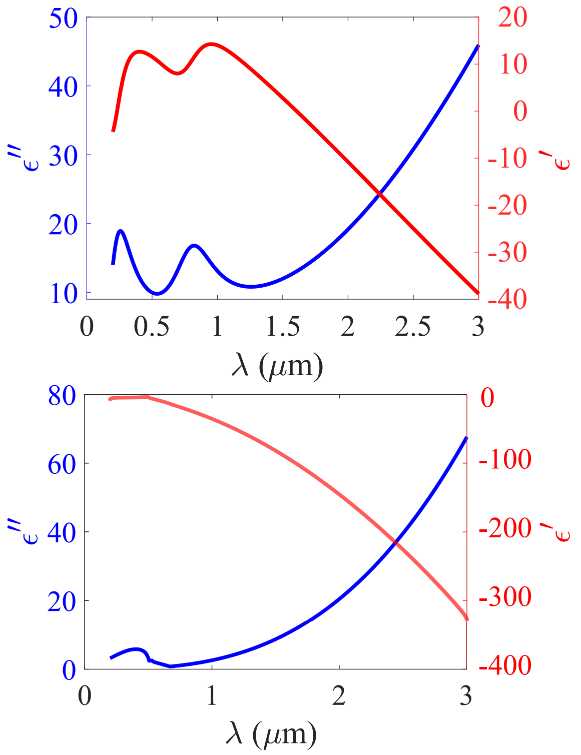

Fig. 1 Dispersion of the dielectric function of (a) titanium carbide[1] and (b) gold as a function of wavelength.

Notes and references

1 K. Chaudhuri, M. Alhabeb, Z. Wang, V. M. Shalaev, Y. Gogotsi and A. Boltasseva, *ACS Photonics*, 2018, **5**, 1115–1122.

[a] *School of Electrical and Computer Engineering, Ben-Gurion University of the Negev, Beer-Sheva 8410501, Israel*
[b] *National Research Tomsk State University, Tomsk, 634050, Russia*
[c] *Tomsk Polytechnic University, Tomsk, 634050, Russia*
§ Email: alinak@bgu.ac.il
